# Multiple Health Outcomes of Daytime Napping: A Comprehensive Umbrella Review

**DOI:** 10.3389/phrs.2026.1609013

**Published:** 2026-02-03

**Authors:** Pengqiang Du, Jiqian Li, Zixin Hua, Yiqi Sun, Siyang Song, Yin Liao, Sheng Cheng, Xingang Li

**Affiliations:** 1 Department of Pharmacy, Fuwai Central China Cardiovascular Hospital, Central China Fuwai Hospital of Zhengzhou University, Zhengzhou, China; 2 Department of Pharmacy, Beijing Friendship Hospital, Capital Medical University, Beijing, China

**Keywords:** all-cause mortality, cardiovascular disease, cognitive impairment, daytime napping, metabolic disease

## Abstract

**Objectives:**

This umbrella review aimed to clarify the dose-response relationship between napping duration and multiple health outcomes.

**Methods:**

Following JBI guidelines, the review included studies from PubMed, Web of Science, the Cochrane Library, and EMBASE. Data on health outcomes, effect sizes, and study characteristics were extracted, and the quality of the studies was assessed using AMSTAR-2 and GRADE. A random effects model and a sensitivity analysis were used to evaluate the associations.

**Results:**

This umbrella review identified 16 meta-analyses encompassing 244 health-related outcomes. Napping for <60 min maximizes cognitive enhancement (SMD = 0.69, 95% CI: 0.37–1.00) and reduces fatigue, while minimizing the risk of all-cause mortality and chronic diseases. Napping for >60 min correlates with a 30% higher risk of coronary heart disease and a 20% increased risk of diabetes and obesity; short naps (20–30 min) improve athletic performance (SMD = 0.99, 95% CI: 0.67–1.31) and recovery, particularly in sleep-deprived individuals.

**Conclusion:**

Limiting nap duration to ≤60 min may optimize cognitive and physical benefits while reducing chronic disease risks. For individuals with chronic conditions, it is prudent to avoid prolonged naps (>60 min) and prioritize nighttime sleep quality.

## Introduction

Napping is a ubiquitous, public health-relevant behavior that accounts for a significant yet understudied part of the daily routine [1, 2]. Despite its high prevalence, the health effects of daytime napping remain controversial: while some evidence supports benefits for cognitive and physical performance, others link napping to adverse health outcomes, such as metabolic disorders [3–6].

Epidemiological data show a global increase in napping frequency and duration, particularly among older individuals, shift workers, and individuals with sleep disorders [7, 8]. However, the relationship between napping and health is complex and shaped by age, gender, and sleep hygiene, and observational studies have reported inconsistent findings [1, 2, 9, 10]. This inconsistency stems from heterogeneous study designs, unstandardized nap duration/timing definitions, and a narrow focus on isolated health domains (e.g., cardiovascular or metabolic outcomes only). Prior systematic reviews and meta-analyses further suffer from three key limitations: 1) the majority focuses on a single health outcome (e.g., cardiovascular disease alone) and neglect neurological function, physical performance, and mental health; 2) many are restricted to specific populations (e.g., older adults or Asian cohorts) and lack generalizability; 3) conflicting conclusions across reviews (e.g., some support the cardiovascular benefits of moderate napping, while others link excessive napping to obesity and diabetes [11, 12]) have not been systematically resolved [13]. These gaps highlight the need for a comprehensive synthesis of existing evidence to clarify the overall nap-health relationship.

Umbrella reviews synthesize findings from existing systematic reviews and meta-analyses, which makes them uniquely positioned to resolve the fragmentation and inconsistency in the current evidence on daytime napping. Notably, few umbrella reviews have addressed sleep-related topics, and even fewer have focused specifically on napping [14–16]. Moreover, existing reviews have synthesized evidence only for cardiac diseases and mortality [16]. While meta-analyses have pooled primary study data, no umbrella review has yet evaluated the nap-health relationship comprehensively across multiple outcomes: cardiovascular, metabolic, neurological, and physical performance, and across all age groups. Against this backdrop, the present umbrella review aims to consolidate existing evidence by integrating and evaluating all relevant systematic reviews, meta-analyses, and the latest quantitative analyses. Our goal is to provide a holistic perspective on nap-related health outcomes across diverse populations, clarify consistent and conflicting findings, and outline the implications for future research and public health initiatives.

## Methods

This umbrella review was conducted according to the Joanna Briggs Institute (JBI) umbrella review guidelines and written according to the Preferred Reporting Items for Overviews of Reviews (PRIOR) statement [13, 17]. The umbrella review protocol was prospectively registered on PROSPERO (CRD42024558520).

### Literature Search

A systematic literature search was conducted in PubMed, Web of Science, the Cochrane Library, and EMBASE for articles that investigated the correlation between daytime napping and health outcomes, with the search date range extending from the inception of the databases to 12 August 2025. The following search terms were used (“napping” OR “siesta” OR “nap” OR “nap sleep” OR “nap time” OR “daytime sleep” OR “daytime nap” OR “daytime napping” OR “day time sleep” OR “day time nap” OR “day time napping” OR “day-time sleep” OR “day-time nap” OR “day-time napping”) AND (“Meta-analyses” OR “Systematic review”). Details of the search strategies are available in Supplementary Table 1.

### Eligibility Criteria

Inclusion criteria: 1) Systematic reviews and meta-analyses with a quantitative synthesis focusing on daytime napping, with original studies including both interventional designs [Randomized Controlled Trials (RCTs) and Non-Randomized Studies of Interventions (NRSIs)] and observational designs (cohort studies and cross-sectional studies); 2) Participants aged 18 years or older, including both general populations and individuals with chronic conditions (e.g., diabetes, cardiovascular diseases); 3) Intervention: Experimental groups exposed to any form of daytime napping; 4) Control groups involving individuals not engaging in any type of daytime napping; 5) Outcomes: Studies reporting quantitative data on multidimensional health outcomes associated with daytime napping, such as cardiovascular diseases, metabolic disorders, physical performance, and neurological performance and so on; 6) Articles written in English; 7) No restrictions on study region or ethnicity.

Exclusion criteria: 1) Conference abstracts, gray literature, protocols, animal studies, meta-analyses and systematic reviews without quantitative analyses; 2) Interventions and experimental groups that involved engaging in napping during nighttime or shift work; 3) Meta-analyses that evaluated the effects of daytime napping on health outcomes in certain disease populations; 4) Literature with a high rate of overlap that is covered by other more recent studies; 5) Articles written in languages other than English.

### Data Extraction

The following information from the research was independently extracted by two authors (JL and ZH): 1) health outcomes (cognitive function, cardiovascular risk, metabolic diseases and other related symptoms) 2) the first author’s name and publication year 3) meta-analysis metrics (nappers vs. non-nappers, long nap vs. non-nap, short nap vs. non-nap, nap following normal sleep vs. non-nap, nap following partial sleep deprivation vs. non-nap, definition of napping, nap duration grouping) 4) estimated effects [relative risk (RR), odds ratio (OR), hazard ratio (HR), Standardized Mean Difference (SMD)], with the 95% confidence intervals (CIs) 5) the number of cohorts/studies, 6) the number of cases/total participants, 7) the study design [cohort, case-control, cross-sectional, randomized controlled trial (RCT), or longitudinal study], 8) the type of effects model (random or fixed), 9) the statistical p- value, 10) I^2^ metric, 11) Cochran’s Q test value, and 12) publication bias (funnel plot visual inspection results and *p*-value of Egger’s test or Begg’s test).

### Overlap Rate Analysis

To determine if there are any overlapping reviews in this umbrella review, an overlap rate analysis was conducted by calculating the covered area (CA) and the corrected covered area (CCA) [18]. The formulas for CA and CCA calculation are presented in Equations 1, 2.

$$CA=\frac{N}{rc}$$ (1)

$$CCA\left(\%\right)=\frac{N-r}{rc-r}\times 100\%$$ (2)

where N is the total number of original studies, r is the number of original studies (excluding overlaps), and c is the number of included reviews [19]. The CCA(%) calculation is divided into the following categories: 1–5 (slight overlap), 6–10 (moderate overlap), 11–15 (high overlap), and >15 (very high overlap) [18].

### Quality Assessment

The methodological quality of the included meta-analyses was assessed using the Assessing the Methodological Quality of Systematic Reviews 2 (AMSTAR-2) tool [20]. The assessment of the quality of evidence for unique outcomes was conducted using the Grading of Recommendations, Assessment, Development, and Evaluation (GRADE) working group classification system [19].

### Data Analyses

The effect sizes (HR, OR, RR, and SMD, and 95% CI) of health outcomes were reanalyzed using random-effects models to compare homogeneous analysis results without changing the original analysis outcome direction [21, 22]. *A p-value* less than 0.05 in two-sided tests was considered statistically significant. Heterogeneity was assessed using I^2^ and the Q test. I^2^ > 50% and *P* < 0.10 indicate a significant heterogeneity. Sensitivity analyses were conducted using the stepwise exclusion method to evaluate the stability of GRADE assessments [23, 24]. For outcomes that could not be reanalyzed, comprehensive analysis results were extracted from the original articles for evaluation. All data analyses were conducted using Stata, version 17.0.

### Stratification of Evidence

A standardized credibility grading system was adopted to systematically assess the included indicators [25, 26]. Evidence was classified into five levels: class I (convincing evidence), class II (highly suggestive evidence), class III (suggestive evidence), class IV (weak evidence), and non-significant [27]. Table 1 shows the criteria for these classifications in detail.

**TABLE 1:** Detailed criteria for the classification of evidence (Worldwide, 2015–2023).

Class	Number of cases	p Value	Remarks
Convincing (class I)	>1,000	<10^−6^	I^2^ < 50% 95% prediction interval excluding the null hypothesis No small-study effects No excess significance bias
Highly suggestive (class II)	>1,000	<10^−6^	Largest study with a statistically significant effect Class I criteria not met
Suggestive (class III)	>1,000	<10^−3^	Class I–II criteria not met
Weak (class IV)	-	<0.05	Class I–III criteria not met
Non-significant	-	>0.05	-

## Results

### Literature and Characteristics of the Included Meta-Analysis

**FIGURE 1:**
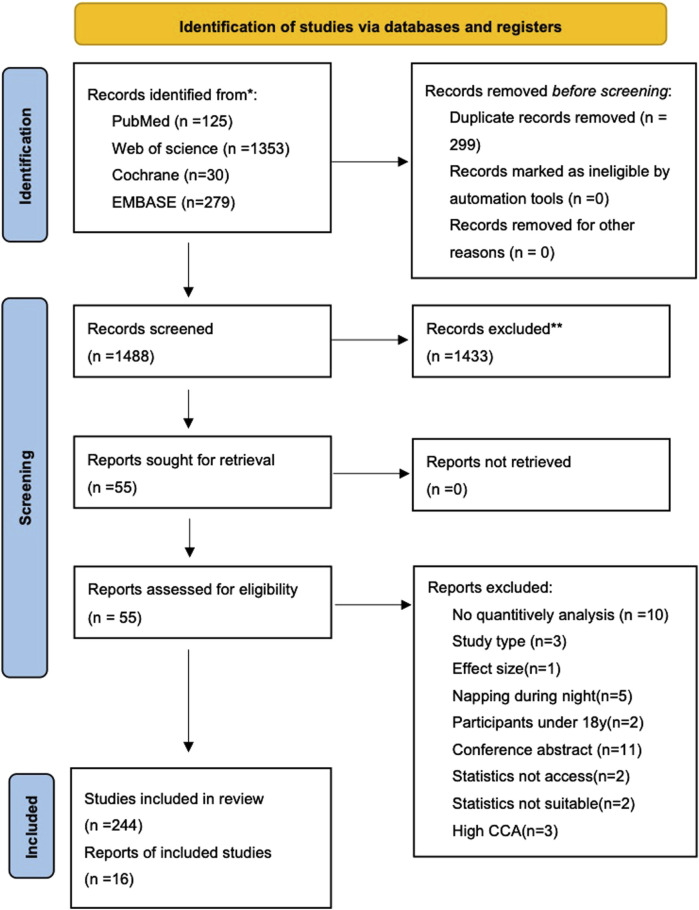
Flowchart of the systematic search and selection process (Worldwide, 2015–2023).

The results of the systematic search and selection of the eligible studies are shown in Figure 1. A total of 1,787 articles were identified, and 299 duplicates were excluded. According to the inclusion and exclusion criteria, 1,488 articles were screened. Finally, 16 meta-analyses were included [28–43], comprising 1 meta-analysis of RCTs [39] and 15 meta-analyses of NRSIs [28–38, 40–43]. Figure 1 shows the screening process. The reasons and exclusion list are shown in Supplementary Table 2.

**FIGURE 2:**
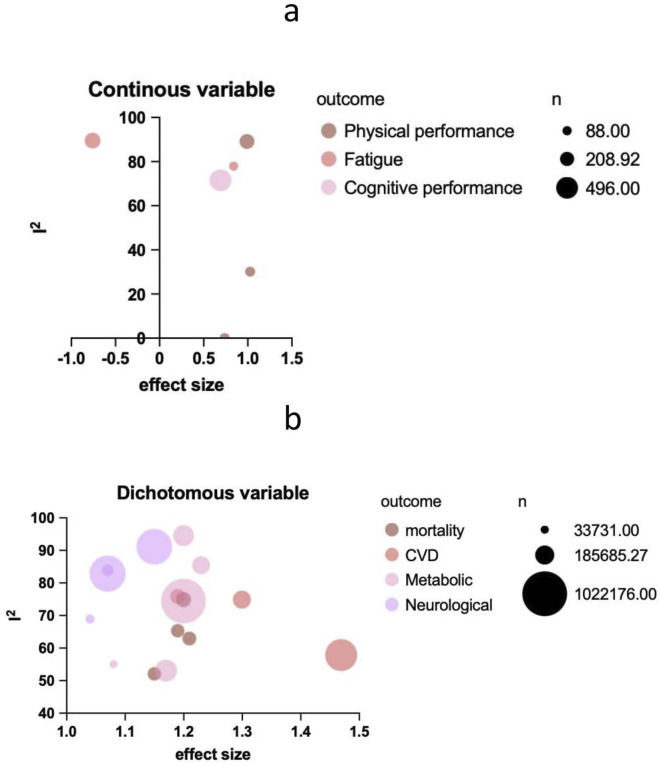
Bubble plot showing multiple health outcomes associated with daytime napping. **(a)** Continuous variable; **(b)** Dichotomous variable (Worldwide, 2015–2023).

All studies included in the review were published after 2015. The majority of the included meta-analyses focused on the associations between daytime napping and metabolic diseases (n = 5), followed by neurological outcomes (n = 4), cardiovascular diseases (n = 3), physical outcomes (n = 2), mortality (n = 2), and cancer (n = 1). Figure 2 synthesizes heterogeneity metrics (I^2^), effect estimates, sample size distributions, and outcome-specific trends via a stratified bubble plot, providing a comprehensive overview of the evidence landscape. The characteristics of the included studies are summarized in Table 2.

**TABLE 2:** Basic statistics of the included meta-analyses (Worldwide, 2015–2023).

Author	Year	Region	Study design	Definition of napping	Nap duration grouping	Follow-up duration	Population	Sample size	Risk of bias rating tools	Outcomes
Celia Alvarez-Bueno	2022	Spain	Cross-sectional [18], longitudinal [7]	Short-duration sleep, typically occurring during the day	NA	0.5–11 years	Adults over 60 years old, women and men	95,719	MMSE; MoCA; TMT; LM-II; COWAT	Cognitive impairment, memory
Wu Fang	2023	China	Cohort [11], cross-sectional [9]	Periods of sleep outside of the main sleep intervals	NA	NA	Adults aged over 38 years old, women and men	1,936,503	NOS	Cognitive impairment/dysfunction, dementia
Liqing Li	2022	China	Cohort [4], cross-sectional [5]	Short-duration sleep during the day	NA	1–17 years	Adults over 18 years old, women and men	649,111	NOS, AHRQ	Depression
Nader Salari	2022	Iran	Cohort [5]Cross-sectional [3]	NA	Grouping: <1 h, ≥1 h; dose-response analysis:15 min	5.1–10 years	Adults over 18 years old, healthy individuals, patients with CHD, patients with orthopedic, ophthalmologic, or urologic issues	167,025	NOS	Risk of coronary heart disease
Wisit Cheungpasitporn	2016	USA	Cross-sectional [9]	NA	NA	NA	Population-based; women and men, adults over 18 years old	112,267	NOS, Cochrane’s Q test	HTN risk
Xiaolin Jin	2020	China	Cohort [7], cross-sectional [1]	NA	NA	5.1–14.3 years	Adults over 25 years old, women and men	524,408	NOS	Stroke (diagnosed or confirmed by death certificate)
Guo-Chong Chen	2017	China	Cohort [7]	NA	<30 min, 31–60 min, >60 min; dose-response analysis: 30 min	5–13 years	Adults aged 30–75 years old	249,077	NA	T2DM
Tomohide Yamada	2015	Japan	Cohort [11]	Short-duration sleep, usually during the day	Long nap: ≥60 min/day; short nap: <60 min/day; dose-response analysis: 0–30 min, 40 min, 90 min	6.3–19 years	Adults over 20 years old (60% women, 40% men)	151,588	NOS	All-cause mortality, cardiovascular disease (fatal and non-fatal)
Vivian Yawei Guo	2017	Hong Kong SAR, China	Cohort [6], cross-sectional [4]	NA	Long nap: ≥60 min/day; short nap: <60 min/day	3–14 years	Adults, with a mean age ranging from 44.3 to 67.3 years old	304,885	NOS	DM
Mengdie Liu	2023	China	Cohort [18], cross-sectional [22]	Short-duration sleep during the day	<30 min; 30–60 min; >60 min	4–14 years	Adults aged 19 years or older, women and men	1,528,216	NA	Diabetes, glycemic control (HbA1c)
Zixin Cai	2023	China	Cohort [5], cross-sectional [7]	NA	NA	NA	Participants from 0 to 88.2 years old, women and men	170,134	NOS	Obesity (BMI)
Guochao Zhong	2015	China	Cohort [12]	NA	Daily napping vs. never napping	4–19 years	Adults, primarily over 65 years old, men and women	130,068	NOS	All-cause mortality, risk of death from CVD, risk of death from cancer
T.C.Erren	2016	Germany	Cohort [20], case-control [4]	NA	With/without napping	1–20 years	Population from Asia, Europe, United States, Australia, women and men	1,500,000	NA	Cancer (registry, database)
Omar Boukhris	2023	Australia	Interventional/prospective [18]	NA	NA	NA	Participants aged 15–35 years old, physically active or an athlete, women and men (primarily men)	269	QualSyst	5-meter shuttle run test (HD, TD, FI), muscle force
Tomohide Yamada	2016	Japan	Cross-sectional [9], cohort [1]	A short nap typically taken during the day	Long nap: ≥60 min/day; short nap: <60 min/day	7–10 years	Western and Asian populations, with a mean age range between 60 and 65 years old, women and men	288,883	NOS	Diabetes, metabolic syndrome
Arthur Eumann Mesas	2022	Spain	Randomized crossover trial [22]	NA	Short nap: <30 min/day; moderate nap: 30–60 min/day; long nap: ≥60 min/day	NA	Male participants aged between 18 and 35 years old, trained athletes, or physically active adults	291	Cochrane Collaboration’s risk of bias 2.0	Cognitive performance, physical performance, fatigue

### Quality Assessment

**FIGURE 3:**
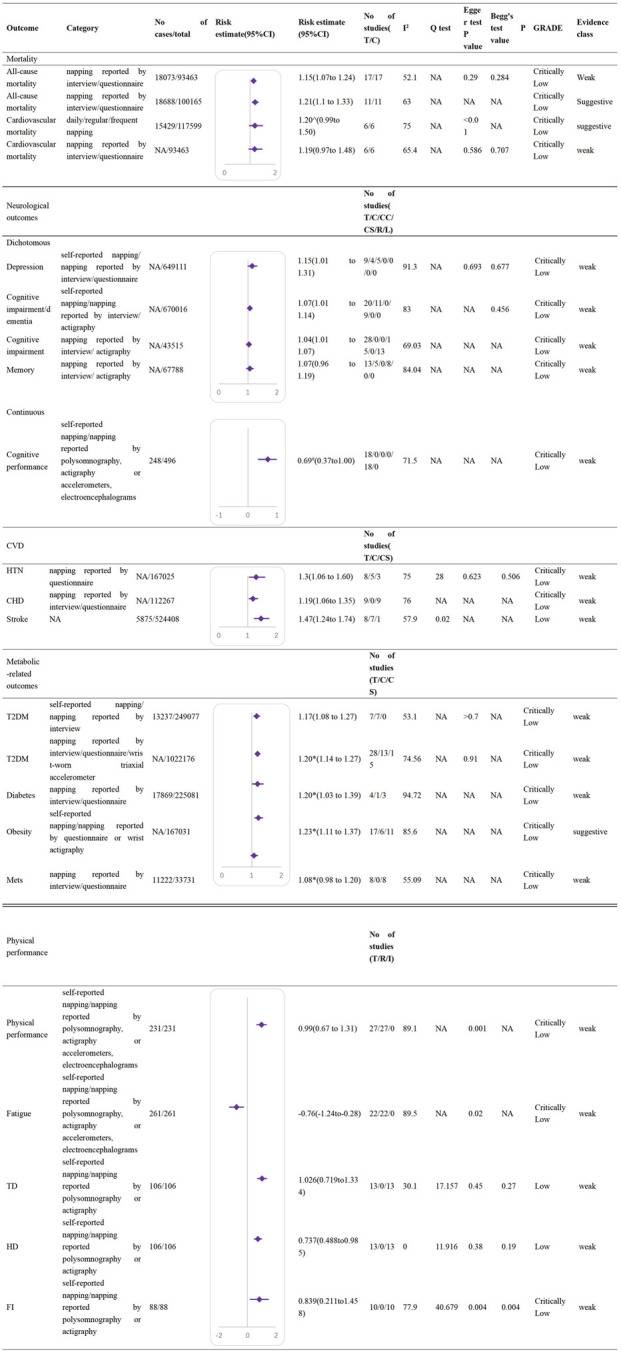
Significant non-dose-response relations between daytime napping and multiple health outcomes (Worldwide, 2015–2023). Note: CI, confidence interval; GRADE, Grading of Recommendations Assessment, Development and Evaluation; NA, not available; CC, case-control studies; R, randomized crossover trials; L, longitudinal studies; HTN: hypertension; CHD, coronary heart disease; CS, cross-sectional studies; T2DM, type 2 diabetes mellitus; Mets, metabolic syndromes; TD, total distance during the 5-m shuttle run test; HD, highest distance during the 5-m shuttle run test; FI, fatigue index during the 5-m shuttle run test; I, interventional prospective studies. *Hazard ratio ^Odds ratio #SMD.

Among the included 16 meta-analyses, two were rated as moderate quality, six as low quality, and eight as critically low according to the AMSTAR2 tool. Due to the majority of the included meta-analyses being observational studies, GRADE classified them as low or critically low quality. The detailed AMSTAR2 and GRADE for each included meta-analysis are available in Supplementary Tables 3, 4. Independent associations were extracted from the 16 meta-analyses, including those on mortality, risk of diabetes, physical performance, and other health outcomes. Credibility grading outcomes indicated that four associations were not statistically significant, 20 associations with P < 0.05 were classified as weak (class IV) evidence, and only three associations were classified as suggestive (class III) evidence. Figure 3 shows the characteristics of the statistically significant associations, while non-significant associations are summarized in Supplementary Tables 5, 6.

### Findings of the Meta-Analyses

#### Mortality

All-cause mortality, cardiovascular mortality, and cancer mortality were estimated. One meta-analysis found that daytime napping is associated with an increased risk of all-cause mortality (HR = 1.22, 95%CI: 1.14–1.31, I^2^ = 42.5%), although no significant associations were observed with cardiovascular mortality (HR = 1.20, 95%CI: 0.99–1.50, I^2^ = 75%) or cancer mortality (HR = 1.07, 95%CI: 0.99–1.15, I^2^ = 8.9%) [43]. A subgroup analysis by nap duration indicated that long naps (>60 min) are associated with higher all-cause mortality (HR = 1.15, 95% CI: 1.04–1.27), while short naps (≤60 min) show no such association (HR = 1.10, 95% CI: 0.92–1.32) [44]. Another subgroup analysis, which was stratified by napping prevalence, showed that a higher risk of all-cause and cardiovascular mortality risk occurs in populations without napping prevalence (HR = 1.23, 95% CI: 1.15–1.32, I^2^ = 36.4%; HR = 1.26, 95% CI: 1.02–1.57, I^2^ = 81.4%), whereas no such associations were found in populations with napping prevalence (HR = 1.23, 95% CI: 0.97–1.57, I^2^ = 55.3%; HR = 1.12, 95% CI: 0.54–2.31, I^2^ = 71.8) [43] (Figure 3).

#### Neurological Outcomes

Four meta-analyses investigating the influence on neurological and cognitive function-related outcomes were included. These comprised 89 studies investigating the relationship between daytime napping and cognitive performance, cognitive impairment, cognitive dysfunction, dementia, memory, and depression. One meta-analysis of 18 randomized controlled trials (RCTs) showed that napping between 12:30 and 16:50 (most commonly at 14:00) following a normal night’s sleep improves cognitive performance (SMD = 0.69, 95% CI: 0.37–1.00, I^2^ = 71.5%) [39]. However, meta-analyses of observational studies reported adverse effects: one meta-analysis found that daily naps longer than 30, 45, or 60 min are associated with an increased risk of cognitive impairment (30 min: OR = 1.35, 95% CI: 1.24–1.48; 45 min: OR = 1.41, 95% CI: 1.27–1.58; 60 min: OR = 1.40, 95% CI: 1.26–1.56), and a pooled analysis indicated an association between napping and dementia (OR = 1.14, 95% CI: 1.07–1.21) [34]. However, another meta-analysis suggested that there is no cross-sectional (global cognition: OR = 1.03, 95% CI: 1.01–1.06; memory: OR = 1.06, 95% CI: 0.90–1.26) or longitudinal (global cognition: OR = 1.00, 95% CI: 0.85–1.18; memory: OR = 1.08, 95% CI: 0.98–1.19) association between napping and specific cognitive functions, including global cognition and memory [28]. A meta-analysis examining the relationship between napping and depression found that daytime napping is associated with an increased risk of depressive symptoms (OR = 1.15, 95% CI: 1.01–1.31, I^2^ = 91.3%) [37] (Figure 3).

#### Cardiovascular Disease

Meta-analyses investigating the relationship between daytime napping and the risk of coronary heart disease, stroke, and hypertension were included. The pooled relative risk (RR) of stroke was 1.47 (95% CI: 1.24–1.74, *P* < 0.001), with significant heterogeneity (I^2^ = 58%, *P* for heterogeneity = 0.02). However, the heterogeneity decreased when the study that did not adjust for sleep duration or stratify the results based on sleep duration was not performed was excluded (RR = 1.38; 95% CI: 1.19–1.60, I^2^ = 44%, *P* for heterogeneity = 0.10) [36]. A meta-analysis comparing nappers with non-nappers reported a pooled RR of 1.19 for hypertension (95% CI: 1.06–1.35) [32]. Another meta-analysis on daytime napping and CHD showed that napping is associated with an increased risk of CHD (RR = 1.30, 95% CI: 1.06–1.60, *P* < 0.001) [40] (Figure 3).

Additionally, a pooled analysis of all included studies demonstrated a 29% increased risk of cardiovascular disease in nappers compared with non-nappers (RR = 1.29, 95% CI: 1.18–1.40).

#### Metabolic-Related Outcomes

Habitual napping was associated with an increased risk of diabetes (OR = 1.20, 95% CI: 1.14–1.27, I^2^ = 74.56%) [38]. A regional subgroup analysis showed a higher diabetes risk in nappers than in non-nappers in Europe (OR = 1.16, 95% CI: 1.03–1.31) and United States (US) (OR = 1.22, 95% CI: 1.08–1.29), but no significant association was observed in the Chinese subgroup (OR = 1.25, 95% CI: 0.86–1.82) [31]. Other subgroup analyses based on nap duration, type of study, gender, and so on indicated a consistently higher risk of diabetes in nappers [35, 45].

Regarding metabolic syndrome, a meta-analysis indicated that long naps (≥60 min) are associated with an increased risk (OR = 1.19, 95% CI: 1.09–1.31) [42].

Regarding obesity, a meta-analysis reported a higher risk in nappers compared with non-nappers (OR = 1.22, 95% CI: 1.10–1.35, I^2^ = 87%) [30], but with variations across countries. A subgroup analysis showed no significant association in Chinese populations (OR = 1.05, 95% CI: 0.90–1.23), whereas significant associations were found in Spain (OR = 9.36, 95% CI: 4.74–18.45), the United States (OR = 1.27, 95% CI: 1.10–1.47), and the United Kingdom (OR = 1.39, 95% CI: 1.32–1.47) [30] (Figure 3).

#### Physical Performance

Two meta-analyses of randomized controlled trials (RCTs) and interventional prospective studies assessed the effects of daytime napping on various physical performance metrics, including 5-meter shuttle run performance, muscle force, fatigue perception, and other related tests. One meta-analysis confirmed that napping between 12:30 and 16:50 (most commonly at 14:00) improves physical performance (SMD = 0.99, 95% CI: 0.67–1.31, I^2^ = 89.1%) and reduces fatigue perception (SMD = −0.76, 95% CI: −1.24 to −0.28; I^2^ = 89.5%) [39]. Another meta-analysis assessed physical performance through the 5-meter shuttle run test: athletes and physically active individuals who napped showed an increased maximum distance (SMD = 1.026, 95% CI: 0.718–1.334, I^2^ = 30.1%) and total distance (SMD = 0.737, 95% CI: 0.488–0.985, I^2^ = 0), along with a decreased fatigue index (SMD = 0.839, 95% CI: 0.211–1.458, I^2^ = 77.9%) [29]. However, no significant effect was found on muscle force (SMD = 0.175, 95% CI: −0.134–0.483, I^2^ = 0). A subgroup analysis suggested higher benefits with a nap duration between 30 and 60 min (physical performance: SMD = 1.74, 95% CI: 1.01–2.46; fatigue: SMD = −1.41, 95% CI: −2.10 to −0.73) and when the interval between nap awakening and testing exceeded 1 h (physical performance: SMD = 1.60, 95% CI: 1.10–2.10; fatigue: SMD = −0.95, 95% CI: −1.51 to −0.38) [39] (Figure 3).

#### Cancer

Only one meta-analysis exploring the relationship between daytime napping and cancer was included. No statistically significant association was found between napping and an increased risk of breast (RR = 0.95, 95%CI: 0.81–1.12, I^2^ = 53.7%) or colorectal cancer (RR = 1.05, 95%CI: 0.98–1.12, I^2^ = 0) [33].

### Sensitivity Analysis

Sensitivity analysis was conducted by sequentially excluding each study, and the pooled results did not show significant changes, indicating that the results of the meta-analyses are robust.

#### Heterogeneity

Among all the included meta-analyses, 11 showed a high degree of heterogeneity (I^2^ ≥ 75%); 13 studies presented a moderate level of heterogeneity (50% < I^2^ < 75%); and 3 studies had low heterogeneity (I^2^ ≤ 50%). Factors contributing to heterogeneity mainly included study design, nap measurement, follow-up duration, and participant characteristics.

#### Publication Bias

Five meta-analyses reported significant publication bias by Egger’s test. Of the included articles, 9 were not linked to a significant publication bias, and 13 did not report the publication bias.

## Discussion

To our knowledge, this is the first umbrella review to comprehensively explore the multifaceted associations between daytime napping and a broad range of health outcomes, including mortality, cardiovascular disease (CVD), diabetes, obesity, neurological function, and physical performance. Daytime napping has become a controversial health topic due to growing attention to its potential adverse effects, and clarifying its health impacts is key to promoting public health.

Our findings indicate that daytime napping is significantly associated with an increased risk of all-cause mortality, although not with cardiovascular mortality [43, 44]. The relationship between napping and neurological outcomes was found to be inconsistent: napping was found to improve cognitive performance, with short-to-moderate naps (<60 min) benefiting cognitive health[39, 46, 47]. Moreover, napping was also closely related to dementia and depression, especially among individuals who nap for extended periods [34, 37]. Additionally, napping was associated with elevated risks of coronary heart disease (19%), hypertension (30%), and stroke (47%) [32, 36, 40]. As for metabolic outcomes, our findings reveal that napping is a significant factor in diabetes, not only increasing the risk of developing the disease but also influencing glycemic control in patients with diabetes. Meanwhile, napping is associated with improved physical performance and reduced perceived fatigue [29, 39]. Notably, evidence on cancer-related outcomes is limited; no significant associations were found between napping and cancer risk or cancer-related mortality [33, 43].

Consistent with our results, previous studies have highlighted an association between nap duration and all-cause mortality: short naps (≤30 min) are associated with lower mortality, while long naps (>60 min) correlate with higher mortality [48, 49]. A cohort study of centenarians in Hainan, China, indicated that male centenarians who nap for at least 2 h during the day have a 97% higher risk of all-cause mortality than those who nap for less time [50]. A meta-analysis of 44 cohort studies further confirmed that habitual napping (especially for >30 min) is associated with an increased risk of all-cause mortality, CVD, and metabolic diseases, with napping for >1 h linked to a 35% higher risk of CVD [51]. Collectively, these studies revealed a positive association between nap duration and mortality risk, wherein the risk increases with longer nap times.

The impact of napping on cognitive function may be mediated by slow-wave activity (δ waves): greater slow-wave activity during napping has been shown to correlate with improved post-nap task performance, supporting memory consolidation and perceptual abilities. Short naps (30 min) have been shown to improve cognitive and behavioral performance, particularly for complex tasks [52]. Nap duration also influences the risk of cognitive impairment: naps less than 30 min are associated with the lowest risk of mild cognitive impairment (MCI), while naps ≥2 h correlate with reduced language fluency, perceptual speed, and overall cognitive performance [53]. These findings highlight the complex, duration-dependent effects of napping on cognitive function.

The mechanisms underlying the association between napping and chronic diseases may involve multiple pathways. For CHD, long-term regular napping may disrupt circadian rhythms, leading to abnormal clock gene expression and exacerbated endothelial dysfunction [54]. A nonlinear relationship was observed between nap duration and hypertension risk: naps ≥90 min were found to be associated with a 1.5-fold higher risk of hypertension, potentially due to delayed nighttime sleep, abnormal diurnal cortisol secretion, and sympathetic hyperactivity [55]. For type 2 diabetes, naps >60 min were found to be linked to a 21% higher risk, possibly mediated by chronic low-grade inflammation with elevated IL-6 and CRP levels and reduced melatonin secretion [56]. Additionally, nap-induced sleep fragmentation may amplify metabolic risks by activating the hypothalamic-pituitary-adrenal axis [57].

Obesity, a trigger for several chronic diseases, such as hyperlipidemia and diabetes, has been found to correlate with napping. The association between napping and obesity may be explained by upregulated IER3 gene expression in nappers (28-fold higher than in non-nappers) [58]. IER3 is overexpressed in the adipose tissue of obese individuals, is promoted by growth factors and inflammatory cytokines, and contributes to the expansion of adipose progenitor cells, chronic inflammation, and hypoxia [59].

The association between napping and physical recovery, along with fatigue relief, has been widely verified by numerous studies. Research has shown that short naps (20–30 min) can significantly alleviate subjective feelings of fatigue, restore physical strength, and improve work performance in the afternoon. The mechanisms underlying these effects may be related to the reduction of cortisol levels and the regulation of neuroendocrine functions [60]. Post-exercise napping has been associated with greater increases in glutathione peroxidase (GPx) and superoxide dismutase (SOD), enhancing antioxidant defense against exercise-induced oxidative stress [61, 62]. For athletes, naps ranging from 25 to 45 min improve physical performance (e.g., grip strength, long jump, and sprint) and reduce muscle soreness, with a 20-min nap benefiting endurance in individuals with insufficient nighttime sleep [2, 63, 64].

Regional variations in the prevalence of daytime napping are substantial and are driven by cultural norms and lifestyle differences. For instance, habitual napping is more common in China, Latin United States, and parts of Europe (e.g., Greece and the Mediterranean regions) than in countries where it is less culturally entrenched [51, 65]. However, the included studies did not explore these regional or ethnic differences in depth, leaving uncertainty about whether such variations may have introduced bias into the pooled results.

After waking up, individuals experience a period of sleep inertia, a transition state characterized by temporary impairments in alertness and cognitive performance following sleep [66]. Sleep inertia can lead to excessive sleepiness and decreased cognitive effort on tasks [67, 68]. Therefore, it might be more appropriate to assess the effects of napping at an appropriate time after sleep inertia has dissipated and cognitive states have stabilized. Evaluating the impact of napping during this stable phase could provide a more objective reflection of its true effects on health. However, determining the optimal timing for assessing napping’s influence on chronic diseases such as hypertension, coronary heart disease, and diabetes remains challenging, as the underlying mechanisms are not yet fully understood, and there is currently no in-depth discussion on this topic in the literature. Additionally, none of the included studies adjusted for sleep conditions (e.g., distinguishing between normal sleep and partial sleep deprivation). Had these studies conducted subgroup analyses based on sleep status, the results might have differed.

Several limitations must be acknowledged. First, methodological flaws in the included meta-analyses may have impacted the reliability of our findings. Specifically, insufficient assessment of publication bias (e.g., failure to conduct a funnel plot analysis or Egger’s test) in some primary reviews could have led to an overestimation of pooled effect sizes. Second, the low overall certainty of the evidence means that the associations between napping and health outcomes may be confounded by unmeasured factors (e.g., nighttime sleep quality, socioeconomic status, and comorbidity severity). Consequently, these observed associations cannot be interpreted as causal relationships, and conclusions should be drawn with caution. Third, the study population is subject to selection bias. Our inclusion criteria restricted the study populations to general adults, excluding studies investigating the effects of napping in children. This limited the generalizability of the results to pediatric populations. Additionally, habitual nappers were overrepresented by older adults, who have a higher baseline risk of cardiovascular and metabolic diseases than younger individuals. Furthermore, research on physical performance was limited to athletes and physically active individuals aged under 35 years of age. These discrepancies mean that findings on physical performance may not apply to inactive populations, particularly older adults, and that the overrepresentation of older adults in disease-related outcomes may have distorted the observed napping-health associations. Fourth, follow-up durations varied across the included studies. Since the health impacts of napping are likely to accumulate over time, inconsistent follow-up periods (e.g., short-term vs. long-term follow-up) may have introduced heterogeneity and compromised the accuracy of assessing napping’s long-term effects. Fifth, some health outcomes lacked sufficient research support. For instance, only three observational studies explored the association between napping and cancer, providing insufficient statistical power to confirm a reliable relationship. Larger-scale, high-quality studies are therefore warranted to clarify these understudied associations.

### Conclusion

This umbrella review provides a comprehensive overview of the impact of napping on health outcomes. For the general population, limiting nap duration to ≤60 min may optimize cognitive and physical benefits while minimizing chronic disease risks. For individuals with chronic conditions (e.g., hypertension or diabetes), it may be advisable to avoid prolonged naps (>60 min) to prioritize nighttime sleep quality. However, due to the low overall quality of the included evidence, these conclusions should be interpreted cautiously. Future research should explore the underlying mechanisms of napping’s health effects, incorporate nighttime sleep quality as a covariate, and conduct subgroup analyses (e.g., by age or baseline health status) to provide more robust evidence for clinical practice and public health policies.
