# Supplementary material for: Multiple Health Outcomes of Daytime Napping: A Comprehensive Umbrella Review
Source: Public Health Rev. 2026 Feb 3;47:1609013. doi: 10.3389/phrs.2026.1609013 (PMC12909254; doi:10.3389/phrs.2026.1609013)
Supplement: Supplementary file 1 [file Supplementaryfile1.zip › supplementary Table1.docx]

Supplementary table1. Search Strategy

PubMed

| search number | query | results |
| --- | --- | --- |
| 1 | ("napping"[Title/Abstract] OR "siesta"[Title/Abstract] OR "nap"[Title/Abstract] OR "nap sleep"[Title/Abstract] OR "nap time"[Title/Abstract] OR "daytime sleep"[Title/Abstract] OR "daytime nap"[Title/Abstract] OR "daytime napping"[Title/Abstract] OR "day time sleep"[Title/Abstract] OR "day time nap"[Title/Abstract] OR "day time napping"[Title/Abstract] OR "day time sleep"[Title/Abstract] OR "day time nap"[Title/Abstract] OR "day time napping"[Title/Abstract]) | 8560 |
| 2 | ("Meta-analyses"[Title/Abstract] OR "systematic review"[Title/Abstract]) | 360486 |
| 3 | #1 AND #2 | 125 |

WOS

| search number | query | results |
| --- | --- | --- |
| 1 | **napping** (All Fields) or **siesta** (All Fields) or **nap** (All Fields) or **nap sleep** (All Fields) or **nap time** (All Fields) or **daytime sleep** (All Fields) or **daytime nap** (All Fields) or **daytime napping** (All Fields) or **day time sleep** (All Fields) or **day time nap** (All Fields) or **day time napping** (All Fields) or **day-time sleep** (All Fields) or **day-time nap** (All Fields) or **day-time napping** (All Fields) | 53500 |
| 2 | **(ALL=(Meta-analyses)) OR ALL=(systematic review)** | 586350 |
| 3 | 1# AND 2# | 1353 |

Embase

| search number | query | results |
| --- | --- | --- |
| 1 | 'napping'/exp OR 'napping' OR 'siesta'/exp OR 'siesta' OR 'nap'/exp OR 'nap' OR 'nap sleep' OR 'nap time' OR 'daytime sleep'/exp OR 'daytime sleep' OR 'daytime nap'/exp OR 'daytime nap' OR 'daytime napping'/exp OR 'daytime napping' OR 'day time sleep' OR 'day time nap' OR 'day time napping' OR 'day-time sleep' OR 'day-time nap' OR 'day-time napping' | 14268 |
| 2 | ('meta analyses' OR systematic) AND ('review'/exp OR review) | 711207 |
| 3 | #1 AND #2 | 279 |

Cochrane Library

| search number | query | results |
| --- | --- | --- |
| 1 | (napping):ti,ab,kw OR (siesta):ti,ab,kw OR (nap):ti,ab,kw OR (nap sleep):ti,ab,kw OR (nap time):ti,ab,kw | 976 |
| 2 | (daytime sleep):ti,ab,kw OR (daytime nap):ti,ab,kw OR (daytime napping):ti,ab,kw OR (day time sleep):ti,ab,kw OR (day time nap):ti,ab,kw | 10137 |
| 3 | (day time napping):ti,ab,kw OR (day-time sleep):ti,ab,kw OR (day-time nap):ti,ab,kw OR (day-time napping):ti,ab,kw | 274 |
| 4 | #1 OR #2 OR #3 | 10758 |
| 5 | ("meta-analyses"):ti,ab,kw OR (systematic review):ti,ab,kw | 29410 |
| 6 | #4 AND #5 | 160(30cochrane review, 130trials) |
