# Supplementary material for: Multiple Health Outcomes of Daytime Napping: A Comprehensive Umbrella Review
Source: Public Health Rev. 2026 Feb 3;47:1609013. doi: 10.3389/phrs.2026.1609013 (PMC12909254; doi:10.3389/phrs.2026.1609013)
Supplement: Supplementary file 1 [file Supplementaryfile1.zip › supplementary Table2.docx]

Supplementary table2. Literature and reasons for exclusion.

| Number | Arthur | Year | Title | Reason |
| --- | --- | --- | --- | --- |
| 1 | Backhaus, W | 2016 | The effect of sleep on motor learning in the aging and stroke population – a systematic review | no quantitive analysis |
| 2 | Chen, J. Y. | 2023 | Causal relationships of excessive daytime napping with atherosclerosis and cardiovascular diseases: a Mendelian randomization study | Mendelian randomization study |
| 3 | Fan, F | 2018 | Daytime napping and cognition in older adults | conference abstract |
| 4 | Farhadian, N | 2021 | The role of daytime napping in declarative memory performance: a systematic review | no quantitive analysis |
| 5 | Guo, V. | 2017 | Long daytime napping over 1 hour per day is associated with increased risk of diabetes | statistics not access |
| 6 | Hilditch, C. J. | 2017 | A review of short naps and sleep inertia: do naps of 30 min or less really avoid sleep inertia and slow-wave sleep? | no quantitive analysis |
| 7 | Lastella, M | 2021 | To Nap or Not to Nap? A Systematic Review Evaluating Napping Behavior in Athletes and the Impact on Various Measures of Athletic Performance | no quantitive analysis |
| 8 | Leong, R. L. F. | 2022 | Systematic review and meta-analyses on the effects of afternoon napping on cognition | effect size: cohen's d |
| 9 | Li, H. | 2019 | Napping on night-shifts among nursing staff: A mixed-methods systematic review | health outcomes resulting from nighttime sleep |
| 10 | Martin-Gill, C. | 2018 | Effects of Napping During Shift Work on Sleepiness and Performance in Emergency Medical Services Personnel and Similar Shift Workers: A Systematic Review and Meta-Analysis | health outcomes resulting from napping during shift work |
| 11 | Mesas, A. E. | 2022 | The role of daytime napping on salivary cortisol in children aged 0–5 years: a systematic review and meta-analysis | Outcomes not suitable for current study |
| 12 | Milner, C. E. | 2009 | Benefits of napping in healthy adults: impact of nap length, time of day, age, and experience with napping | no quantitive analysis |
| 13 | Pan, Z | 2020 | The association between napping and the risk of cardiovascular disease and all-cause mortality: A systematic review and dose-response meta-analysis | conference abstract |
| 14 | Patterson, P. D. | 2021 | Does the evidence support brief (≤30 mins), moderate (31–60 mins), or long duration naps (61+ mins) on the night shift? A systematic review | health outcomes resulting from nighttime sleep |
| 15 | Qiao, X. Q. | 2024 | Excessive Daytime Napping Increases the Risk of Non-Alcoholic Fatty Liver Disease: A Meta-Analysis and a Mendelian Randomization Study | include Mendelian Randomization Study |
| 16 | Ruggiero,J. S. | 2014 | Effects of Napping on Sleepiness and Sleep-Related Performance Deficits in Night-Shift Workers: A Systematic Review | health outcomes resulting from nighttime sleep |
| 17 | Sirohi, P | 2022 | A systematic review of effects of daytime napping strategies on sports performance in physically active individuals with and without partial-sleep deprivation | no quantitive analysis |
| 18 | Soesanto, S. A. | 2024 | The Impact of Mid Day Nap Duration on Blood Pressure: A Systematic Review and Meta-Analysis | not access |
| 19 | Souabni, M | 2021 | Benefits of Daytime Napping Opportunity on Physical and Cognitive Performances in Physically Active Participants: A Systematic Review | no quantitive analysis |
| 20 | Souabni, M | 2022 | Benefits and risks of napping in older adults: A systematic review | no quantitive analysis |
| 21 | Sun, J. H. | 2022 | Daytime napping and cardiovascular risk factors, cardiovascular disease, and mortality: A systematic review | an umbrella review |
| 22 | Thorpe, K | 2015 | Napping, development and health from 0 to 5 years: A systematic review | no quantitive analysis |
| 23 | Tomohide, Y | 2016 | Daytime napping and the risk of metabolic diseases: dose-response meta-analysis | conference abstract |
| 24 | Wannamethee, S. G. | 2024 | Napping and Obesity in Adults – What do we Know? | no quantitive analysis |
| 25 | Yamada, T | 2014 | Day napping and risk of type 2 diabetes: a dose-response meta-analysis | conference abstract |
| 26 | Yamada, T | 2015 | Excessive Daytime Sleepiness, Daytime Napping, and Risk of Type 2 Diabetes: A Meta-analysis | conference abstract |
| 27 | Yamada, T | 2016 | Daytime Napping, Daytime Sleepiness and the Risk of Metabolic Diseases: Dose-Response Meta-analysis Using Restricted Cubic Spline Model | conference abstract |
| 28 | Yamada, T | 2015 | Excessive daytime sleepiness, daytime napping, and risk of type 2 diabetes: a meta-analysis | conference abstract |
| 29 | Yamada, T | 2016 | J-Shaped Relationship between Nap Time and the Risk of Diabetes or Metabolic Syndrome: Dose-Response Meta-analyses Using Restricted Cubic Spline Models | conference abstract |
| 30 | Yan, B | 2017 | GW28-e0565 Daytime napping durations and the incidence of cardiovascular diseases: a prospective cohort study | conference abstract |
| 31 | Yan, B | 2017 | A prospective cohort study on the relationship between daytime nap and stroke | conference abstract |
| 32 | Yan, B | 2017 | Siesta may contribute to coronary artery disease: A prospective observational study | conference abstract |
| 33 | Dutheil, F | 2020 | Napping and cognitive performance during night shifts: a systematic review and meta-analysis | health outcomes resulting from nighttime sleep |
| 34 | Li J. X. | 2023 | Daytime Napping and Cognitive Health in Older Adults: A Systematic Review | no quantitive analysis |
| 35 | Frederic Dutheil | 2021 | Effects of a Short Daytime Nap on the Cognitive Performance: A Systematic Review and Meta-Analysis | the formats of data are not suitable for analysis |
| 36 | Ruihua Liu | 2017 | Age- and gender-specific associations of napping duration with type 2  diabetes mellitus in a Chinese rural population: the RuralDiab study | excluded due to a high CCA |
| 37 | Xiaokun Liu | 2015 | Meta-Analysis of Self-Reported Daytime  Napping and Risk of Cardiovascular or All-Cause  Mortality | excluded due to a high CCA |
| 38 | Andressa Alves da Silva | 2015 | Sleep duration and mortality in the elderly: a systematic review with meta-analysis | excluded due to a high CCA |
| 39 | Zhe Pan | 2020 | Association of napping and all-cause mortality and incident cardiovascular diseases: a dose-response meta-analysis of cohort studies | Statistics not available |
