# Supplementary material for: Multiple Health Outcomes of Daytime Napping: A Comprehensive Umbrella Review
Source: Public Health Rev. 2026 Feb 3;47:1609013. doi: 10.3389/phrs.2026.1609013 (PMC12909254; doi:10.3389/phrs.2026.1609013)
Supplement: Supplementary file 1 [file Supplementaryfile1.zip › Supplementary Table4.docx]

Supplementary table4. GRADE scoring criteria for complete associations of daytime napping with health outcomes.

| **Source, no. of studies (Design)** | **Risk of bias** | **Inconsistency** | **Indirectness** | **Imprecision** | **Publication bias** | **quality** |
| --- | --- | --- | --- | --- | --- | --- |
| Mortality | | | | | | |
| Guochao Zhong, 9 | slightly risk | no serious risk | no serious risk | no serious risk | undetected | Low |
| Guochao Zhong, 4 | serious risk | slightly risk | no serious risk | slightly risk | undetected | Critically Low |
| Guochao Zhong, 5 | slightly risk | no serious risk | no serious risk | no serious risk | undetected | Low |
| Xiaokun Liu, 3 | serious risk | unclear | no serious risk | slightly risk | undetected | Critically Low |
| Xiaokun Liu, 3 | slightly risk | unclear | no serious risk | no serious risk | undetected | Critically Low |
| Xiaokun Liu, 6 | serious risk | slightly risk | no serious risk | no serious risk | undetected | Critically Low |
| Tomohide Yamada， 11 | serious risk | slightly risk | no serious risk | no serious risk | undetected | Critically Low |
| Guochao Zhong, 6 | serious risk | serious risk | no serious risk | slightly risk | undetected | Critically Low |
| Guochao Zhong, 3 | serious risk | slightly risk | no serious risk | slightly risk | undetected | Critically Low |
| Guochao Zhong, 3 | slightly risk | serious risk | no serious risk | no serious risk | undetected | Critically Low |
| Guochao Zhong, 4 | serious risk | no serious risk | no serious risk | slightly risk | undetected | Critically Low |
| Cardiovascular disease | | | | | | |
| Wisit Cheungpasitporn, 8 | serious risk | serious risk | no serious risk | slightly risk | slightly risk | Critically Low |
| Nader Salari, 8 | serious risk | serious risk | no serious risk | no serious risk | undetected | Critically Low |
| Nader Salari, 6 | serious risk | no serious risk | no serious risk | no serious risk | undetected | Low |
| Nader Salari, 7 | serious risk | slightly risk | no serious risk | no serious risk | undetected | Critically Low |
| Nader Salari, 2 | serious risk | no serious risk | no serious risk | no serious risk | undetected | Low |
| Nader Salari, 3 | serious risk | no serious risk | no serious risk | no serious risk | undetected | Low |
| Nader Salari, 3 | serious risk | serious risk | no serious risk | no serious risk | undetected | Critically Low |
| Xiaolin Jin, 8 | slightly risk | slightly risk | no serious risk | no serious risk | undetected | Low |
| Xiaolin Jin, 2 | slightly risk | no serious risk | no serious risk | no serious risk | undetected | Low |
| Xiaolin Jin, 3 | slightly risk | no serious risk | no serious risk | no serious risk | undetected | Low |
| Xiaolin Jin, 2 | slightly risk | no serious risk | no serious risk | slightly risk | undetected | Low |
| Metabolic-related outcomes | | | | | | |
| Guo-Chong Chen, 7 | serious risk | slightly risk | no serious risk | no serious risk | undetected | Critically Low |
| Guo-Chong Chen, 1 | slightly risk | no serious risk | no serious risk | no serious risk | undetected | Low |
| Guo-Chong Chen, 4 | serious risk | no serious risk | no serious risk | no serious risk | undetected | Low |
| Guo-Chong Chen, 2 | slightly risk | no serious risk | no serious risk | slightly risk | undetected | Critically Low |
| Tomohide Yamada, 5 | slightly risk | no serious risk | no serious risk | no serious risk | undetected | Low |
| Tomohide Yamada, 5 | serious risk | serious risk | no serious risk | slightly risk | undetected | Critically Low |
| Vivian Yawei Guo, 3 | slightly risk | no serious risk | no serious risk | no serious risk | undetected | Low |
| Vivian Yawei Guo, 6 | serious risk | slightly risk | no serious risk | slightly risk | undetected | Critically Low |
| Mengdie Liu, 28 | serious risk | slightly risk | no serious risk | no serious risk | undetected | Critically Low |
| Tomohide Yamada, 8 | serious risk | slightly risk | no serious risk | slightly risk | undetected | Critically Low |
| Tomohide Yamada, 4 | slightly risk | no serious risk | no serious risk | no serious risk | undetected | Low |
| Tomohide Yamada, 4 | serious risk | no serious risk | no serious risk | slightly risk | undetected | Critically Low |
| Zixin Cai, 20 | serious risk | serious risk | no serious risk | no serious risk | undetected | Critically Low |
| Zixin Cai, 10 | serious risk | serious risk | no serious risk | slightly risk | undetected | Critically Low |
| Zixin Cai, 2 | slightly risk | no serious risk | no serious risk | no serious risk | undetected | Low |
| Zixin Cai, 6 | serious risk | serious risk | no serious risk | no serious risk | undetected | Critically Low |
| Zixin Cai, 2 | slightly risk | no serious risk | no serious risk | no serious risk | undetected | Low |
| Neurological performance | | | | | | |
| Arthur Eumann Mesas, 32 | serious risk | slightly risk | no serious risk | no serious risk | slightly risk | Critically Low |
| Celia Alvarez-Bueno, 27 | serious risk | slightly risk | no serious risk | no serious risk | slightly risk | Critically Low |
| Celia Alvarez-Bueno, 21 | serious risk | slightly risk | no serious risk | slightly risk | slightly risk | Critically Low |
| Celia Alvarez-Bueno, 6 | serious risk | slightly risk | no serious risk | slightly risk | slightly risk | Critically Low |
| Wu Fang, 20 | serious risk | serious risk | no serious risk | no serious risk | undetected | Critically Low |
| Wu Fang, 3 | serious risk | serious risk | no serious risk | slightly risk | undetected | Critically Low |
| Wu Fang, 8 | serious risk | no serious risk | no serious risk | no serious risk | undetected | Low |
| Wu Fang, 4 | serious risk | slightly risk | no serious risk | slightly risk | undetected | Critically Low |
| Wu Fang, 7 | serious risk | no serious risk | no serious risk | no serious risk | undetected | Low |
| Wu Fang, 9 | serious risk | slightly risk | no serious risk | slightly risk | undetected | Critically Low |
| Wu Fang, 6 | serious risk | no serious risk | no serious risk | no serious risk | undetected | Low |
| Wu Fang, 14 | serious risk | serious risk | no serious risk | slightly risk | undetected | Critically Low |
| Wu Fang, 7 | serious risk | no serious risk | no serious risk | no serious risk | undetected | Low |
| Celia Alvarez-Bueno, 14 | serious risk | no serious risk | no serious risk | slightly risk | slightly risk | Critically Low |
| Celia Alvarez-Bueno, 11 | serious risk | serious risk | no serious risk | slightly risk | slightly risk | Critically Low |
| Celia Alvarez-Bueno, 3 | serious risk | slightly risk | no serious risk | slightly risk | slightly risk | Critically Low |
| Liqing Li, 9 | serious risk | serious risk | no serious risk | no serious risk | undetected | Critically Low |
| Physical Performance | | | | | | |
| Arthur Eumann Mesas, 27 | slightly risk | serious risk | no serious risk | slightly risk | slightly risk | Critically Low |
| Arthur Eumann Mesas, 9 | slightly risk | slightly risk | no serious risk | slightly risk | slightly risk | Critically Low |
| Arthur Eumann Mesas, 10 | slightly risk | serious risk | no serious risk | slightly risk | slightly risk | Critically Low |
| Arthur Eumann Mesas, 4 | slightly risk | slightly risk | no serious risk | slightly risk | slightly risk | Critically Low |
| Arthur Eumann Mesas, 6 | slightly risk | slightly risk | no serious risk | slightly risk | slightly risk | Critically Low |
| Arthur Eumann Mesas, 11 | slightly risk | serious risk | no serious risk | slightly risk | slightly risk | Critically Low |
| Omar Boukhris, 13 | serious risk | no serious risk | no serious risk | no serious risk | undetected | Low |
| Omar Boukhris, 13 | slightly risk | no serious risk | no serious risk | no serious risk | undetected | Low |
| Omar Boukhris, 11 | serious risk | no serious risk | no serious risk | slightly risk | slightly risk | Critically Low |
| Arthur Eumann Mesas, 22 | slightly risk | serious risk | no serious risk | slightly risk | slightly risk | Critically Low |
| Arthur Eumann Mesas, 8 | slightly risk | serious risk | no serious risk | serious risk | slightly risk | Critically Low |
| Arthur Eumann Mesas, 8 | slightly risk | serious risk | no serious risk | slightly risk | slightly risk | Critically Low |
| Arthur Eumann Mesas, 2 | slightly risk | serious risk | no serious risk | serious risk | slightly risk | Critically Low |
| Arthur Eumann Mesas, 4 | slightly risk | serious risk | no serious risk | serious risk | slightly risk | Critically Low |
| Arthur Eumann Mesas, 11 | slightly risk | serious risk | no serious risk | slightly risk | slightly risk | Critically Low |
| Omar Boukhris, 10 | serious risk | serious risk | no serious risk | no serious risk | no serious risk | Critically Low |

Evaluation Criteria：

|  | No serious risk/undetected(0) | Slightly risk(-1) | Serious risk(-2) |
| --- | --- | --- | --- |
| Risk of bias | More than 2/3 of the original research is low-risk | More than 2/3 of the original research has moderate risk  Or  The results of observational studies are consistent | More than 2/3 of the original research is high-risk  Or  Inconsistent observational research results |
| Inconsistency | I^2^≤50%  （I^2^ is high but the result direction is the same, it will not be downgraded） | 50%<I^2^<75%, | I^2^≥75% |
| Indirectness | The meta-analyses included in this study is completely related to the research question and does not involve this issue, so it will not be downgraded | | |
| Imprecision | 1. 95% CI did not cross the equivalent line  And  2. Total sample size included in all studies: categorical variables ≥ 300 cases, continuous variables ≥ 400 cases | 1. 95% CI did cross the equivalent line  Or  2. Total sample size included in all studies: categorical variables < 300 cases, continuous variables <400 cases | 1. 95% CI did cross the equivalent line  2. Total sample size included in all studies: categorical variables < 300 cases, continuous variables <400 cases |
| Publication bias | Not meeting both of the following conditions simultaneously: 1) accepting vendor sponsorship, 2) asymmetric funnel plot | Meeting both of the following conditions simultaneously: 1) accepting vendor sponsorship, 2) asymmetric funnel plot | / |

GRADE classification standard：

|  | High | Moderate | Low | Very |
| --- | --- | --- | --- | --- |
| Total score of RCT | ≥0 | -1 | -2 | ≤-3 |
| Total score of observational study | ≥2 | 1 | 0 | ≤-1 |
