# Supplementary material for: Multiple Health Outcomes of Daytime Napping: A Comprehensive Umbrella Review
Source: Public Health Rev. 2026 Feb 3;47:1609013. doi: 10.3389/phrs.2026.1609013 (PMC12909254; doi:10.3389/phrs.2026.1609013)
Supplement: Supplementary file 1 [file Supplementaryfile1.zip › Supplementary Table5.docx]

Supplementary Table 5. Associations between daytime napping and mortality

| **Source** | **Outcome** | **Category** | **No of cases/total** | **MA metric** | **Risk estimate**  **(95%CI)** | **No of studies(Cohort)** | **Follow-up(y)** | **Effects model** | **I^2^** | **Q test** | **Egger test P value** | **Begg's test P value** |
| --- | --- | --- | --- | --- | --- | --- | --- | --- | --- | --- | --- | --- |
| **Significant** | | | | | | | | | | | | |
| Xiaokun Liu | All-cause mortality | napping reported by interview/questionnaire | 18073/93463 | RR | 1.15(1.07 to 1.24) | 17 | 6.9-20 | RE | 52.1 | NA | 0.29 | 0.284 |
| Tomohide Yamada | All-cause mortality | napping reported by interview/questionnaire | 18688/100165 | RR | 1.21(1.1 to 1.33) | 11 | 7.8-20 | RE | 63 | NA | NA | NA |
| Guochao Zhong | Cardiovascular mortality | daily/regular/frequent napping | 15429/117599 | HR | 1.2*(0.99 to 1.50) | 6 | 4-14.3 | RE | 75 | NA | <0.01 | NA |
| Xiaokun Liu | Cardiovascular mortality | napping reported by interview/questionnaire | NA/93463 | RR | 1.19(0.97 to 1.48) | 6 | 6.9-14.3 | RE | 65.4 | NA | 0.586 | 0.707 |
| **Non-significant** | | | | | | | | | | | | |
| Guochao Zhong | All-cause mortality | napping reported by interview/questionnaire | 18926/106387 | HR | 1.22(1.14 to 1.31) | 9 | 4-19 | RE | 42.5 | NA | NA | 0.08 |
| Guochao Zhong | Cancer-related mortality | napping reported by interview/questionnaire | 14891/92059 | HR | 1.07(0.99 to 1.15) | 4 | 6.5-14.3 | RE | 8.9 | NA | NA | >0.05 |

MA, meta-analysis; CI, confidence interval; NA, not available; RR, relative risk; HR, hazard ratio; RE, random effect model
