# Supplementary material for: Multiple Health Outcomes of Daytime Napping: A Comprehensive Umbrella Review
Source: Public Health Rev. 2026 Feb 3;47:1609013. doi: 10.3389/phrs.2026.1609013 (PMC12909254; doi:10.3389/phrs.2026.1609013)
Supplement: Supplementary file 1 [file Supplementaryfile1.zip › Supplementary Table6.docx]

Supplementary Table 6. Associations between daytime napping and physical performance

| **Source** | **Outcome** | **Category** | **No of cases/total** | **MA metric** | **Risk**  **estimate(95%CI)** | **No of studies(T/R/I)** | **Effects model** | **I^2^** | **Q test** | **Egger test P value** | **Begg's test P value** |
| --- | --- | --- | --- | --- | --- | --- | --- | --- | --- | --- | --- |
| **Significant** | | | | | | | | | | | |
| Arthur Eumann Mesas | Physical performance | self-reported napping/napping reported by polysomnography, actigraphy or accelerometers, electroencephalograms | 231/231 | SMD | 0.99(0.67 to 1.31) | 27/27/0 | RE | 89.1 | NA | 0.001 | NA |
| Arthur Eumann Mesas | Fatigue | self-reported napping/napping reported by polysomnography, actigraphy or accelerometers, electroencephalograms | 261/261 | SMD | -0.76(-1.24 to -0.28) | 22/22/0 | RE | 89.5 | NA | 0.02 | NA |
| Omar Boukhris | TD | self-reported napping/napping reported by polysomnography or actigraphy | 106/106 | SMD | 1.026(0.719 to 1.334) | 13/0/13 | RE | 30.1 | 17.157 | 0.45 | 0.27 |
| Omar Boukhris | HD | self-reported napping/napping reported by polysomnography or actigraphy | 106/106 | SMD | 0.737(0.488 to 0.985) | 13/0/13 | RE | 0 | 11.916 | 0.38 | 0.19 |
| Omar Boukhris | FI | self-reported napping/napping reported by polysomnography or actigraphy | 88/88 | SMD | 0.839(0.211 to 1.458) | 10/0/10 | RE | 77.9 | 40.679 | 0.004 | 0.004 |
| **Non-significant** | | | | | | | | | | | |
| Omar Boukhris | MF | self-reported napping/napping reported by polysomnography or actigraphy | 21/21 | SMD | 0.175(-0.134 to 0.483) | 11/0/11 | RE | 0 | 2.95 | 0.002 | 0.34 |

MA, meta-analysis; CI, confidence interval; NA, not available; TD, total distance during the 5-m shuttle run test; HD, highest distance during the 5-m shuttle run test; FI, fatigue index during the 5-m shuttle run test; MF, muscle force; T, total number of studies; R, randomized crossover trial; I, interventional prospective studies; SMD, standard mean difference.
